# Exosomes secreted by adipose-derived mesenchymal stem cells regulate type I collagen metabolism in fibroblasts from women with stress urinary incontinence

**DOI:** 10.1186/s13287-018-0899-9

**Published:** 2018-06-13

**Authors:** Xiaochun Liu, Shiwei Wang, Suhui Wu, Qian Hao, Ying Li, Zhuodan Guo, Wenzhen Wang

**Affiliations:** 1 Department of O/G, Shanxi Academy of Medical Sciences & Shanxi Da Yi Hospital, Shanxi Da Yi Hospital Affiliated to Shanxi Medical University, Taiyuan, 030032 China; 2 School of Medicine, University of Tasmania, Hobart, 7000 Australia

**Keywords:** Mesenchymal stem cells, Exosomes, Col1a1, Collagen metabolism

## Abstract

**Background:**

Mesenchymal stem cells (MSC) have gained credibility as a therapeutic tool partly due to their potential to secrete factors such as cytokines and chemokines. Recently, exosomes, which mediate intercellular communication by delivering biomolecules such as mRNA and miRNA into recipient cells, have gained attention as a new and valuable therapeutic strategy in regenerative medicine. However, the potential role of exosomes secreted by adipose-derived mesenchymal stem cells (adMSC-Exos) in collagen metabolism is not well understood. The purpose of this study was to evaluate the effects of adMSC-Exos on collagen metabolism in cultured fibroblasts from women with stress urinary incontinence (SUI).

**Methods:**

Periurethral vaginal wall tissues of postmenopausal women with or without SUI were collected during transvaginal surgical procedures. Primary fibroblasts were cultured from periurethral vaginal wall tissues, and the levels of type I collagen mRNA and protein were examined by qRT-PCR and western blotting. MSC were isolated from human adipose tissue by enzymatic digestion. Exosomes were prepared by ultracentrifugation of adMSC-conditioned medium (adMSC-CM) and were confirmed by transmission electron microscopy and western blot analyses. The effects of adMSC-CM and adMSC-Exos were assessed using qRT-PCR and western blotting.

**Results:**

The type I collagen content was significantly decreased in periurethral vaginal wall tissues and cultured vaginal fibroblasts from women with SUI. adMSC-CM increased the expression of the col1a1 gene in vaginal fibroblasts from women with SUI. adMSC-Exos could be successfully isolated from adMSC-CM and could be transferred to fibroblasts efficiently. adMSC-Exos increased the expression of col1a1 in vaginal fibroblasts from women with SUI, and when the fibroblasts were treated with adMSC-Exos, the expression levels of TIMP-1 and TIMP-3 in fibroblasts were upregulated, with significant downregulation of MMP-1 and MMP-2 expression levels.

**Conclusions:**

adMSC-Exos increased type I collagen contents by increasing collagen synthesis and decreasing collagen degradation in vaginal fibroblasts from women with SUI. adMSC-Exos may be a novel therapeutic approach for treating SUI.

## Background

Stress urinary incontinence (SUI), the most common form of incontinence, is defined as involuntary leakage of urine with effort or exertion, such as coughing or sneezing [1]. SUI is a major health problem affecting a large number of women of reproductive and menopausal ages and imposes a significant financial burden on health systems worldwide [2, 3]. The underlying pathology and cause of SUI remain unknown, but many studies suggest that the development of SUI in women is accompanied by fibroblast dysfunction and reduced collagen [4–8].

Surgical treatment is the current gold standard therapy for SUI, and short-term success has been achieved with injectable bulking agents [9, 10]. However, complications and poor long-term efficacy cause patients to seek alternative treatments, especially those that may provide safer and longer-lasting outcomes for SUI [11–13]. Recently, stem cell therapy has been extensively investigated at the experimental level and in a variety of clinical applications [14, 15]. Given its ability to induce tissue regeneration, stem cell-based treatment represents a promising strategy to overcome the limitations of current treatments for SUI.

Mesenchymal stem cells (MSC) are currently the most advanced and safe cell therapy tool for several diseases because of their easy isolation, high expansion potential resulting in an unlimited pool of transplantable cells, low immunogenicity, amenability to ex vivo genetic modification, and multipotency [16–19]. During the past 10 years, many pre-clinical and clinical studies on stem cells for SUI treatment have been published. For example, Zou et al. developed a tissue-engineered sling with bone marrow-derived mesenchymal stem cells seeded in a degradable silk scaffold and found that the tissue-engineered sling showed convincing functional effects for the treatment of stress urinary incontinence in a rat model [20]. Zhou et al. found that implantation of ADSCs via urethral/intravenous injection significantly decreased the abnormal voiding rate of an SUI rat model compared to the control group [21]. Lee et al. reported that intrinsic sphincter deficiency and mixed stress incontinency were effectively improved in 10 of 39 women with various types of stress urinary incontinence after transurethral umbilical cord blood stem cell injection [22]. All the results above indicate the regenerative potential of MSC for the treatment of SUI, but our understanding of how MSC mediate their effects on the continence mechanism is limited.

MSC are classically thought to exert a tissue repair effect through their capacity for multilineage differentiation and self-renewal. Indeed their regeneration and repair effects have been mainly attributed to their secreted factors rather than their multilineage differentiation capacity [23, 24]. Exosomes which are defined as nanosized membrane vesicles with a diameter of 30–100 nm have been recently isolated from MSC-conditioned medium and these stem cell-derived exosomes are suggested to be the key intercellular communication mediators between MSC and target cells and to be associated with numerous physiological and pathological functions [25–28]. Here, to better understand the exosome-mediated intercellular communication between MSC and fibroblasts from women with SUI relative to collagen metabolism we first isolated and characterized human adipose-derived MSC (adMSC-Exos). Then we investigated the roles of exosomes secreted by adMSC-Exos in collagen metabolism. We observed that adMSC-Exos can promote the proliferation of fibroblasts. Furthermore advanced studies have shown that adMSC-Exos promoted collagen synthesis and inhibited collagen degradation by modulating the expression levels of TIMP metallopeptidase inhibitor 1 (TIMP-1) matrix metallopeptidase 1 (MMP-1) and type I collagen. To the best of our knowledge, this is the first report to show that adMSC-Exos can regulate collagen metabolism in fibroblasts from women with SUI. Because adMSC-Exos upregulate the concentration of type I collagen in fibroblasts from women with SUI they may represent a potential treatment candidate for SUI.

## Methods

### Tissue collection

Periurethral vaginal wall tissues were obtained from SUI(+) (*n* = 12) and SUI(−) (*n* = 12) patients. A signed informed consent form was obtained from all participating women. Women with a history of endometriosis, gynecologic malignancies, connective tissue disorders, prior pelvic surgery, and advanced pelvic organ prolapse (higher than stage II on the Pelvic Organ Prolapse Quantification system) were excluded. Approximately 1 cm^2^ of full-thickness periurethral vaginal wall was excised 1 cm lateral to the urethrovesical junction from women undergoing surgery for SUI. Biopsies (1 cm^2^) of vaginal walls from a similar area were excised from controls undergoing transvaginal gynecologic surgery.

### Cell culture

We isolated fibroblasts from vaginal wall tissues and cultured them as described previously [29]. The purity of cultured fibroblasts was confirmed by immunofluorescence using mouse anti-alpha smooth muscle actin antibody monoclonal antibody (1:1000; Abcam, Cambridge, MA, USA). Fibroblasts isolated from each patient’s vaginal wall tissue were cultured in 6-well plates (BD Falcon, Franklin Lakes,NJ,USA) in triplicate. Cells from passage 3 were used in the experiments.

Adipose tissue was obtained from human liposuction aspirates with informed consent from the donors (healthy women and SUI patients). Isolation of adMSC was performed as previously reported [30]. Human adMSC were resuspended in the culture medium and seeded at a density of 2 × 10^6^ cells per dish (10 cm). Cultures were maintained in a 37 °C incubator with 5% CO2 and passaged with trypsin/ethylenediaminetetraacetic acid when the cells were 70% confluent.

### Collagen synthesis assay

The percent of collagen synthesized (as a percent of total protein) was determined [31]. The cells were washed and incubated for 3 h at 37 °C with 2,3-^3^H-proline in medium containing ascorbic acid (50 mg/mL). Cells and media were collected, and the protein was precipitated overnight with 20% trichloroacetic acid (TCA) at 4 °C. The precipitate was collected by centrifugation at 3000 rpm for 10 min, and the obtained pellet was washed three times with 5% TCA/0.01% proline. The resulting pellet was dissolved in 0.2 M NaOH, and the solution was titrated to a pH of 7.6 with 0.2 M HCl. This solution was divided into two aliquots. One aliquot was digested with 100 mL of collagenase (2 mg/mL; Sigma-Aldrich, St Louis, MO, USA). The other aliquot was incubated in the buffer (without collagenase) for 1 h at 37 °C. Proteins were precipitated with 10% TCA and 0.5% tannic acid for 1 h on ice. The precipitate was separated from the supernatant by centrifugation at 10,000 rpm for 5 min. The supernatants were retained, and the pellets were dissolved in 0.2 M NaOH. Radioactivity in the supernatants and the dissolved pellets was determined by liquid scintillation counting. Synthesized collagen was considered a collagenase releasable count (supernatant of the collagenase-treated aliquot). Total protein was the value of collagenase releasable counts plus collagenase insensitive counts (pellet of the collagenase-treated aliquot). A correction factor of 5.4 was applied to the collagenase insensitive counts to correct for the relative abundance of proline and hydroxyproline. Collagen synthesis is reported as a percent of the total protein synthesized.

### Western blot analysis

Sodium dodecyl sulfate (SDS) sample buffer (0.5 M Tris-HCl, pH 6.8, 20% sucrose, 10% SDS, 1% bromophenol blue) was added to the concentrated samples. After equal amounts of protein (5 μg/lane) were separated on 4–20% gradient polyacrylamide gels (Bio-Rad, Hercules, CA, USA) under nonreducing conditions, the gels were blotted onto nitrocellulose membranes (Pierce, Waltham, MA, USA) in an electrophoretic transfer cell (Bio-Rad). Blots were blocked with 5% non-fat milk at 4 °C overnight and then probed with Anti-Collagen I antibody (1:1000; Abcam, Cambridge, UK, ab23446), anti-CD63 antibody (1:1000; Abcam, ab59479), anti-Hsp70 antibody (1:1000; Abcam, ab2787) and anti-CD81 antibody (1:1000; Abcam, ab59477) at room temperature for 1 h. After three washes with PBS-T (pH 7.4 and 0.1% Triton), the membrane was incubated with sheep anti-mouse IgG conjugated to HRP (NeoBioscience, Shenzhen, China) for 1 h at room temperature, followed by three washes in PBS-T. The band density was determined by Bio-Rad Quality One Software.

### Real-time RT–PCR

Total RNA was extracted with a Total RNA Isolation Kit (Takara Bio, Shiga, Japan) according to the manufacturer’s instructions and quantified using spectrophotometry (NANO drop 2000; Thermo Fisher Scientific, Waltham, MA, USA). The primer sequences were: Col1a1, 5’-GAGGGCCAA GACGAAGACATC-3′ (sense) and 5’-CAGATCACGTCATCGCACAAC-3′ (anti-sense); MMP-1, 5’-AAAATTACACGCCAGATTTGCC-3′ (sense) and 5′-GGT GTGACATTACTCCAGA GTTG-3′ (anti-sense); MMP-2, 5’-GATACCCCTTTGA CGGTAAGGA-3′ (sense) and 5’-CCTTCTCCCAAGGTCCATAGC-3′ (anti-sense); MMP-9, 5’-AGACCTGGGCAGATTCCAAAC-3′ (sense) and 5’-CGGCAAGTCTT CCGAGTAGT-3′ (anti-sense); TIMP-1, 5’-AGAGTGTCTGCGGATACTTCC-3′ (sense) and 5’-CCAACAGTGTAGGTCTTGGTG-3′ (anti-sense); TIMP-2, 5’-GCTG CGAGTGCAAGATCAC-3′ (sense) and 5’-TGGTGCCCGTTGATGTTCTTC-3′ (anti-sense); TIMP-3, 5’-CATGTGCAGTACATCCATACGG-3′ (sense) and 5’-CATC ATAGACGCGACCTGTCA-3′ (anti-sense). The relative expression of mRNAs was evaluated by the 2^–ΔΔCt^ method and normalized to the expression of GAPDH. The cycling program involved preliminary denaturation at 95 °C for 1 min, followed by 45 cycles of denaturation at 95 °Cfor 10 s, annealing at 62 °Cfor 25 s, and elongation at 62 °C for 20 s, followed by a final elongation step at 70 °C for 5 min.

### Immunofluorescence staining

Fibroblasts were fixed at room temperature with 4% PFA for 10 min. After permeabilization in 1% Triton X-100/PBS for 15 min, nonspecific binding was blocked with 3% bovine serum albumin for 1 h at 37 °C. Then, fibroblasts were incubated in mouse anti-alpha smooth muscle actin antibody monoclonal antibody (1:1000; Abcam, ab119952) at the appropriate dilution at 4 °C overnight. Secondary antibodies were used for 1 h at 37 °C after washing with PBS. Hoechst counterstain was used for visualization. Pictures were captured using an Olympus inverted fluorescence microscope (Olympus, Tokyo, Japan).

### Flow cytometric analysis of cell immunophenotype

For immunophenotype analysis of adMSC, cells were trypsinized and washed with 2 mL of phosphate-buffered saline (PBS) containing 0.5% bovine serum albumin (BSA; Sigma) per tube. Cells were counted and centrifuged at 1200 g for 5 min. After discarding the supernatant, the cell pellet was resuspended in 50 μL of PBS containing fluorescein isothiocyanate (FITC)-labeled primary antibodies and incubated at 4 °C for 30 min. FITC-labeled immunoglobulin G1 (IgG1) of the same species and isotypes was used as negative control. Finally, the cells were washed twice with 2 mL of PBS containing 0.5% BSA and resuspended in 0.5 mL of PBS for fluorescence-activated cell sorting (FACS) analysis. The working concentrations of the primary antibodies against human CD29 (eBioscience, San Diego, CA, USA; 11–0299-42), CD31 (eBioscience; 11–0319-42), CD34 (eBioscience; 11–0349-42), CD44 (eBioscience; MA1–10228), CD105 (eBioscience; MA1–19594) and HLA-DR (eBioscience; 11–9956-42) were 10–20 ng/mL. Flow cytometric analysis was performed with a flow cytometer BD Accuri™ C6 (BD Biosciences, Franklin Lakes, NJ, United States). Data were analyzed with CFlow Plus 1.0 (BD Biosciences).

### Multilineage differentiation of adMSC

To identify the capacity for adMSC multilineage differentiation, adMSCs were cultured under differentiation conditions. For adipocyte differentiation, adMSCs were cultured at 2–3 10^4^/cm^2^ in DMEM with 10% FCS, 10^− 6^ M dexamethasone, 50 mg/mL ascorbic acid, and 100 mg/mL 1-methyl-3-isobutyl-xanthine (all from Sigma-Aldrich). After 14 days, Oil Red O staining was performed to show adipocyte differentiation. For osteoblast differentiation, adMSCs were cultured at 10^4^/cm^2^ in DMEM with 10% FBS, 10 mM β-glycerophosphate, 10^− 7^ M dexamethasone, and 0.2 mM ascorbic acid (all from Sigma-Aldrich). After 21 days, the cells were stained with ALP staining to reveal osteogenic differentiation.

### Exosome extraction

Exosome extraction was performed as previously described [32]. AdMSC-Exos were generated from adMSCs of healthy women, and SUI-Exos were generated from adMSCs of SUI patients. Briefly, adMSCs were cultured for 48 h before exosome isolation. Then, the medium was harvested and centrifuged at 800 g for 5 min, followed by 2000 g for 10 min to remove the lifted cells. The supernatants were subjected to filtration on a 0.1-mm-pore polyethersulfone membrane filter (Corning, Corning, NY, USA) to discard large vesicles and cell debris. Then, the mixtures were concentrated by a 100,000-Mw cutoff membrane (Centricon^®^ Plus-70, Merck Millipore, Burlington, MA, USA). The volume of the supernatants was reduced from approximately 250–500 mL to 30 mL. Then, the supernatants were ultracentrifuged at 100,000 g for 1 h at 4 °C using a 70Ti rotor (Beckman Coulter, Brea, CA, USA). Exosomes were then resuspended in 6 mL of PBS and ultracentrifuged at 100,000 g for 1 h at 4 °C using a 100Ti rotor (Beckman Coulter).

### Dil-labeled exosomes to fibroblasts

Purified adMSC-Exos were labeled with 1 μM of Dil (Invitrogen, Waltham, MA, USA) as previously described [33]. Pelleted exosomes were washed to remove unbound Dil, resuspended in PBS/5% BSA, and then added to fibroblast medium for 6 h. Fibroblasts were then washed in PBS, fixed in 4% paraformaldehyde, and imaged by microscopy.

### Transmission electron microscopy

Purified exosomes were fixed with 1% glutaraldehyde in PBS (pH 7.4). After rinsing, a 20-μL drop of the suspension was loaded onto a formvar/carbon-coated grid negatively stained with 3% (*w*/*v*) aqueous phosphotungstic acid for 1 min for observation by transmission electron microscopy (Hitachi, Tokyo, Japan, SU-8010).

### Statistical analysis

All values are shown as the mean ± s.d. Differences between two groups were determined with the unpaired Student’s *t* test. ANOVA was used for multiple comparisons. *P* < 0.05 was considered significant.

## Results

### Collagen concentration in periurethral vaginal wall tissues and cultured vaginal fibroblasts from women with or without SUI

**Fig. 1:**
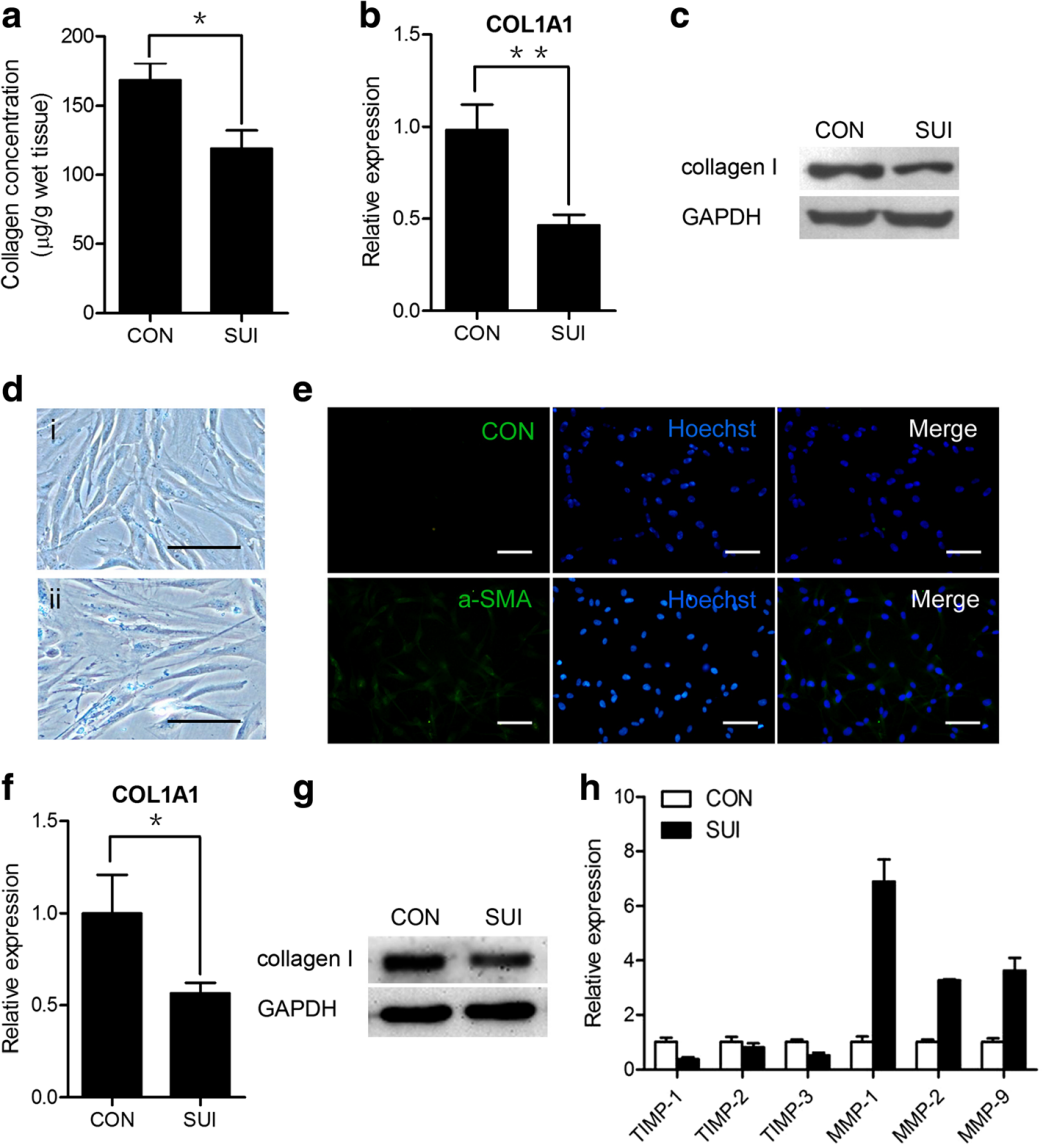
Collagen concentration in periurethral vaginal wall tissues and cultured vaginal fibroblasts from women with or without SUI. **a** Total collagen concentration measured as micrograms of hydroxyproline per milligrams of wet weight from urogenital tissues of women with SUI and in urologically healthy women. The results are the mean ± s.d. (*n* = 12 for each group). *P < 0.05. **b** The levels of Col1a1 in urogenital tissues of women with SUI and in urologically healthy women were analysed by qRT-PCR. **P < 0.01. **c** The expression of type I collagen in urogenital tissues was determined by a western blot assay. **d** The morphology of cultured vaginal fibroblasts in women with SUI (ii) and in urologically healthy women (i). **e** ɑ-SMA protein expression by immunofluorescence staining (*green*). Original scale bars = 50 μm. **f** The levels of Col1a1 in cultured vaginal fibroblasts from women with or without SUI were analyzed by qRT-PCR. The results are the mean ± s.d. (*n* = 3 for each group). *P < 0.05. **g** The expression of type I collagen in cultured vaginal fibroblasts was determined by a western blot assay. **h** The levels of TIMP-1, TIMP-2, TIMP-3, MMP-1, MMP-2, and MMP-9 in fibroblasts from women with or without SUI were analyzed by qRT-PCR. The results are the mean ± s.d. (*n* = 3 for each group). CON control, *MMP-1* matrix metallopeptidase 1, *MMP-3* matrix metallopeptidase 3, *MMP-9* matrix metallopeptidase 9, *SUI* stress urinary incontinence, *TIMP-1* TIMP metallopeptidase inhibitor 1, *TIMP-2* TIMP metallopeptidase inhibitor 2, *TIMP-3* TIMP metallopeptidase inhibitor 3

To investigate collagen expression levels in periurethral vaginal wall tissues of postmenopausal women with or without SUI, vaginal wall tissues were collected from women with SUI during transvaginal surgical procedures (Table 1). The total collagen concentration in periurethral vaginal wall tissues from women with SUI was significantly downregulated compared to that in the tissues from women without SUI (*P* < 0.05; Fig. 1a). qRT-PCR analysis showed that the expression levels of Col1a1 were significantly reduced in the women with SUI (*P* < 0.01; Fig. 1b). Western blotting also demonstrated that type I collagen was significantly reduced in the patients with SUI (Fig. 1c).

**Table 1:** Case description

	With SUI	Without SUI	*P* value
	(*n* = 12)	(*n* = 12)	
Age, yrs	63.08 ± 5.93	62.50 ± 8.15	0.843
Parity, n	2.67 ± 0.89	2.50 ± 0.80	0.633
Mode of labor	Vaginal	Vaginal	
Birth weight, g	3345.83 ± 542.49	3283.22 ± 518.45	0.776
BMI, kg/m2	31.07 ± 2.83	29.33 ± 3.60	0.201
Menopausal duration, yrs	8.42 ± 2.19	8.50 ± 2.39	0.930

The data of age, parity, birth weight, body mass index (BMI) and menopause duration of 12 patients in each group were compared, and no differences were detected

Collagen is mainly synthesized by fibroblasts in periurethral vaginal wall tissue. To examine collagen metabolism changes in fibroblasts from women with SUI, fibroblasts were isolated from periurethral vaginal wall tissues of women with SUI and from healthy women. We found that cultured vaginal fibroblasts from the women with SUI showed more elongation than those from the healthy women (Fig. 1d). Immunostaining assay identified the fibroblast marker ɑ-smooth muscle actin (ɑ-SMA) in these fibroblasts as expected (Fig. 1e). qRT-PCR analysis showed that the expression of Col1a1 was significantly reduced in the patients with SUI (Fig. 1f). Western blotting also demonstrated that type I collagen was significantly reduced in the patients with SUI (Fig. 1g). Furthermore, we found that fibroblasts from the women with SUI exhibited significant decreases in the expression levels of TIMP-1, TIMP-2 and TIMP-3, while the mRNA expression levels of MMP-1, MMP-2, and MMP-9, which reportedly increase collagen degradation in stress incontinence, were significantly increased (Fig. 1h). Taken together, these data suggested that the type I collagen content was significantly decreased in periurethral vaginal wall tissues and cultured vaginal fibroblasts from the women with SUI.

### Characterization of human adMSC

**Fig. 2:**
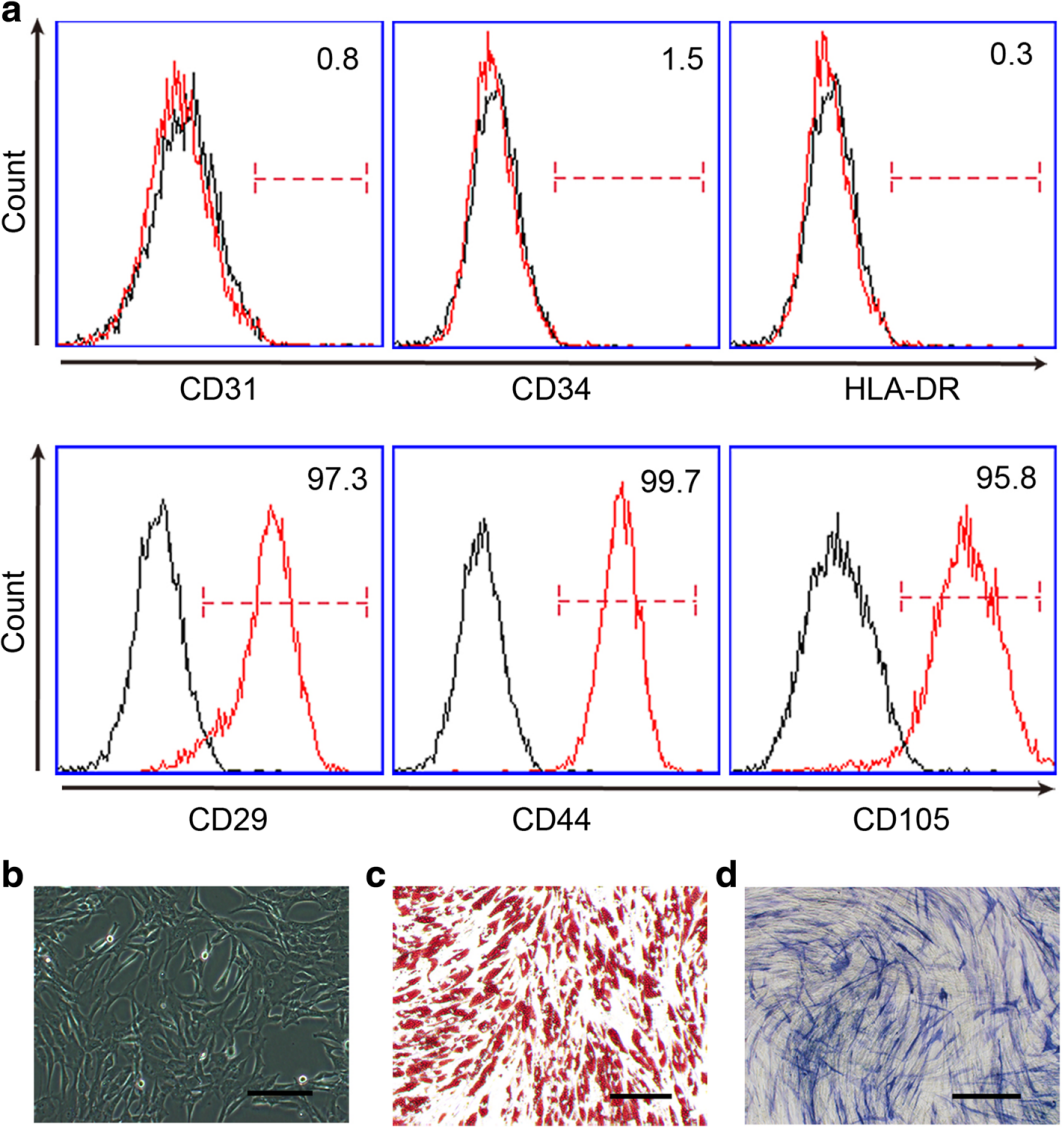
Characterization of human adMSC. **a** Immunophenotype of human adMSC. **b** The morphology of human adMSC was observed under light microscopy. Original scale bars = 50 μm. **c** The differentiation capacity of human adMSC was demonstrated by Oil red O staining for adipocytes (**c**) and ALP staining for osteoblasts (**d**). Original scale bars = 50 μm

MSC were initially isolated from the bone marrow; however, the isolation of bone marrow MSC requires invasive aspiration from a donor, significantly restricting its application. Because adipose tissue is another rich source of MSC (adMSC) and enables auto-grafting, adipose tissue has recently become a popular source for MSC isolation. In this study, to study the effects of MSC on the collagen metabolism of fibroblasts, we isolated MSC from human adipose tissues. We found that human adMSC have a characteristic morphology of fibroblast-like cells (Fig. 2b). They express high levels of CD29, CD44, and CD105, but they are persistently negative for CD31, CD34, and HLA-DR (Fig. 2a) as previously reported [30]. Under appropriate culture conditions, adMSC can differentiate into adipocytes and osteoblasts as demonstrated by Oil red O staining and ALP staining, respectively (Fig. 2c, d).

### adMSC-conditioned medium regulates type I collagen metabolism in cultured vaginal fibroblasts from women with SUI

**Fig. 3:**
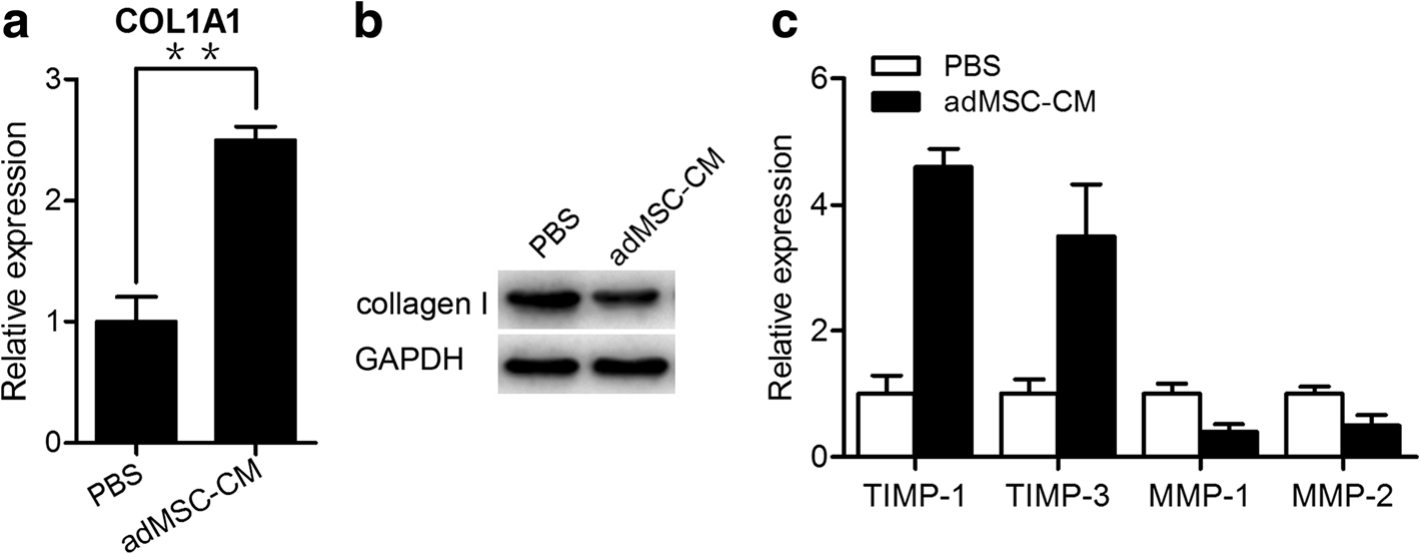
adMSC-conditioned medium regulates type I collagen metabolism in cultured vaginal fibroblasts from women with SUI. **a** Fibroblasts were incubated with adMSC-conditioned medium for 48 h. The mRNA levels of col1a1 were evaluated by qRT-PCR. The results are the mean ± s.d. (*n* = 3 for each group). **P < 0.01. **b** The expression of type I collagen was determined by a western blot assay. **c** The mRNA levels of TIMP-1, TIMP-3, MMP-1, and MMP-2 were evaluated by qRT-PCR. The results are the mean ± s.d. (*n* = 3 for each group). *adMSC-CM* adipose-derived mesenchymal stem cell-conditioned medium, *MMP-1* matrix metallopeptidase 1, *MMP-3* matrix metallopeptidase 3, *TIMP-1* TIMP metallopeptidase inhibitor 1, *TIMP-2* TIMP metallopeptidase inhibitor 2

To study the effect of adMSC on collagen metabolism, fibroblasts from women with SUI were treated with adMSC-conditioned medium for 48 h. qRT-PCR and western blotting analysis showed that the expression of Col1a1 in fibroblasts from the women with SUI was significantly increased after treatment with adMSC-conditioned medium (Fig. 3a, b). Furthermore, when the fibroblasts were treated with adMSC-conditioned medium, we found that the expression levels of TIMP-1 and TIMP-3 were significantly increased, while the mRNA expression levels of MMP-1 and MMP-2, which reportedly decrease collagen degradation in stress incontinence, were significantly decreased (Fig. 3c). Therefore, our results indicated that adMSC-conditioned medium regulated collagen metabolism by increasing collagen synthesis and decreasing collagen degradation in vaginal fibroblasts in women with SUI.

### Isolation and characterization of adMSC-Exos

**Fig. 4:**
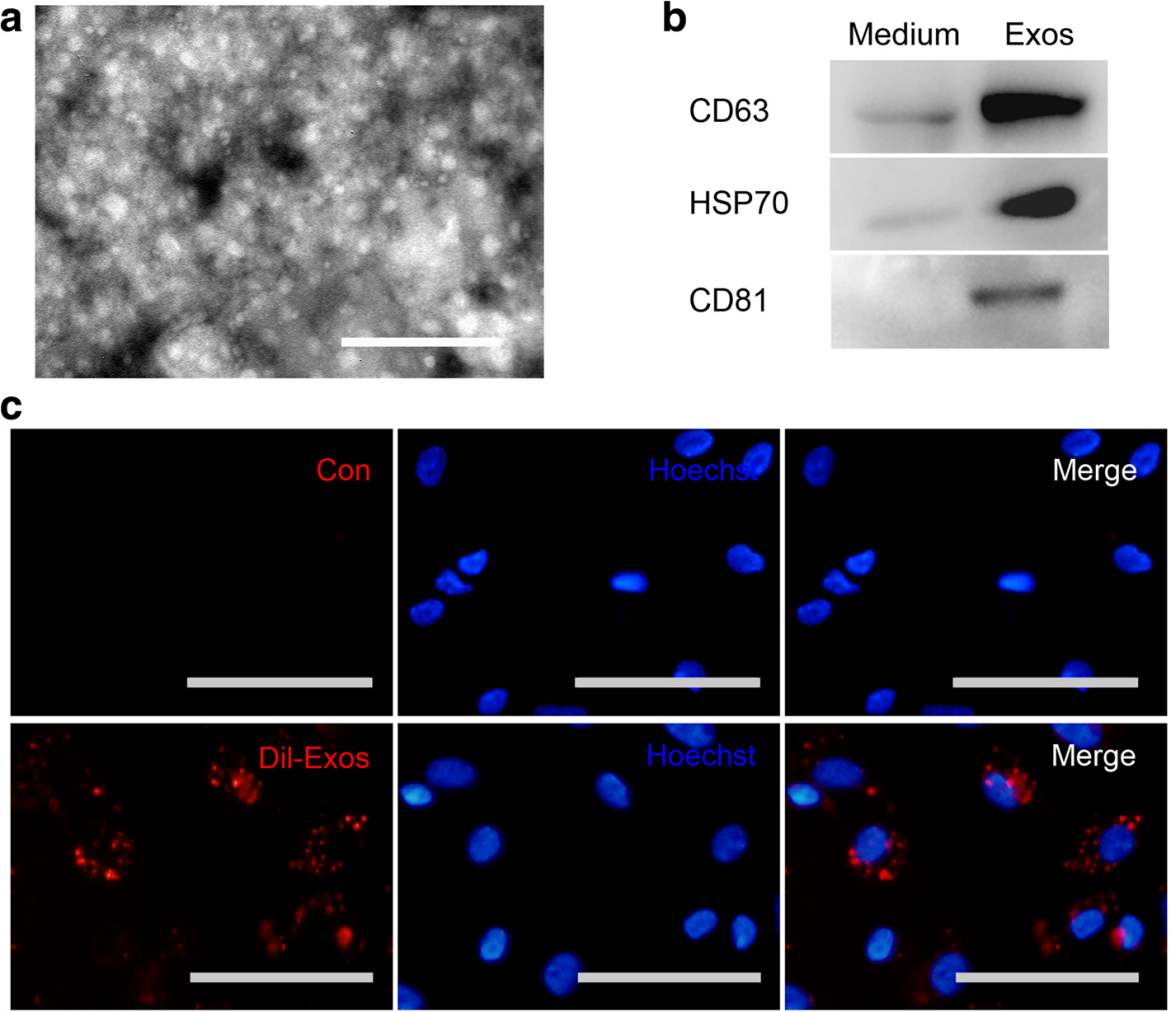
Isolation and characterization of adMSC-Exos. **a** Electron micrograph of exosomes isolated from adMSC-conditioned medium. The *arrowheads* indicate exosomes. Original scale bars = 0.5 μm. **b** Western blotting was performed with adMSC-Exos (*Exos*) or adMSC-conditioned medium (*Medium*). CD63, HSP70 and CD81 expression levels in exosomes were detected. **c** Fibroblasts were incubated with Dil-labeled exosomes (*Dil-Exos*; Dil is shown in *red*) or carrier control (*CON*), and nuclei were stained with Hoechst 33,342 (*blue*). Original scale bars = 50 μm

To study the roles of exosomes in collagen metabolism, adMSC-Exos were first isolated and characterized as previously described [33]. Transmission electron microscopy analysis showed that the exosomes purified from the adMSC-conditioned medium were round membrane-bound vesicles with a size ranging from 30 to 100 nm in diameter (Fig. 4a). Western blot demonstrated that the exosome marker proteins CD63, HSP70, and CD81 were present in adMSC-Exos as expected (Fig. 4b). To further investigate whether the adMSC-Exos could be transferred to fibroblasts, adMSC-Exos were labeled with Dil dye and incubated with fibroblasts from women with SUI in vitro. The uptake was confirmed by fluorescence microscopy. After 8 h, more than 90% of the fibroblasts were Dil-positive; the Dil-labeled adMSC-Exos had been taken up and were transferred to cytoplasm compartments (Fig. 4c). These data suggested that adMSC-Exos were successfully isolated and could be efficiently transferred to fibroblasts.

### adMSC-Exos regulate type I collagen metabolism in cultured vaginal fibroblasts from women with SUI

**Fig. 5:**
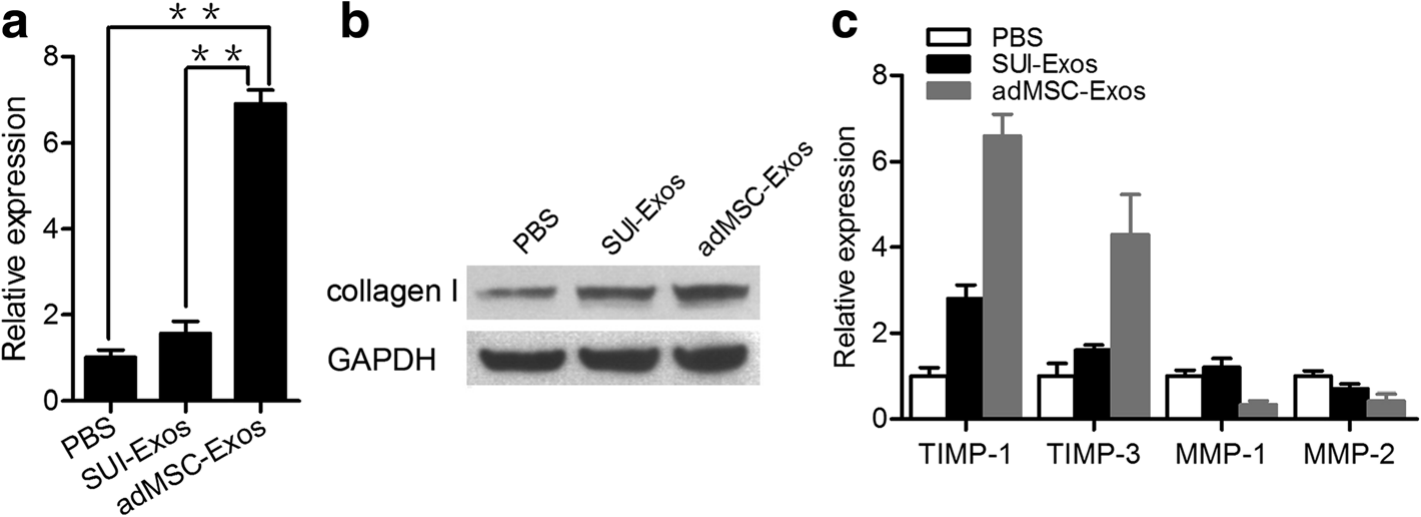
adMSC-Exos regulate type I collagen metabolism in cultured vaginal fibroblasts from women with SUI. Fibroblasts were incubated with adMSC-Exos or SUI-Exos for 48 h. **a** The levels of Col1a1 in cultured vaginal fibroblasts from women with SUI were analyzed by qRT-PCR. The results are the mean ± s.d. (*n* = 3 for each group). **P < 0.01. **b** The expression of type I collagen was determined by a western blot assay. **c** The levels of TIMP-1, TIMP-3, MMP-1, and MMP-2 in fibroblasts from women with SUI were analyzed by qRT-PCR. The results are the mean ± s.d. (*n* = 3 for each group). *adMSC-Exos* exosomes secreted by adipose-derived mesenchymal stem cells, *MMP-1* matrix metallopeptidase 1, *MMP-3* matrix metallopeptidase 3, *SUI* stress urinary incontinence, *TIMP-1* TIMP metallopeptidase inhibitor 1, *TIMP-2* TIMP metallopeptidase inhibitor 2

To examine the effects of adMSC-Exos on collagen metabolism, fibroblasts from women with SUI were treated with exosomes for 48 h. Our results showed that the expression levels of col1a1 were increased in the fibroblasts incubated with adMSC-Exos compared to those of the fibroblasts incubated with SUI-Exos (Fig. 5a, b). Moreover, when the fibroblasts were treated with adMSC-Exos, the expression levels of TIMP-1 and TIMP-3 in the fibroblasts were upregulated, with significant downregulation of MMP-1 and MMP-2 (Fig. 5c). Taken together, our observations suggested that adMSC-Exos increased the type I collagen content by increasing collagen synthesis and decreasing collagen degradation in vaginal fibroblasts from women with SUI.

## Discussion

Stress urinary incontinence (SUI), which is defined as involuntary leakage of urine during an increase in abdominal pressure, is a prevalent urological problem that is most common in women, with an incidence of 18.9% in those aged > 20 years in China [34]. The cause of stress urinary incontinence is multifactorial. Previous studies have reported a decreased collagen content and altered morphologic features in the pelvic support tissues of women with stress urinary incontinence [7, 8]. In the present study, we found that the expression of type I collagen in periurethral vaginal wall tissues of postmenopausal women with SUI was significantly decreased compared to that in tissues from women without SUI, which is consistent with the observations of Chen et al., who demonstrated a 60% lower collagen content in the vaginal wall tissues of women with SUI compared to that in tissues from age-matched continent women [35].

Mesenchymal stem cells (MSC), which have the capacity for self-renewal and differentiation into chondrocytes, osteoblasts, and adipocytes, can be isolated and easily expanded from tissues such as bone marrow, adipose tissue, the umbilical cord, placenta, dental pulp and others [36, 37]. Here, we isolated MSC from human adipose tissues, adMSC, expressing cell surface markers such as CD29, CD44, and CD105, without the expression of other markers, including CD31, CD34, and HLA-DR surface molecules, and showing differentiation potential into osteocytes and adipocytes under in vitro conditions. It has been increasingly observed in various fields of regenerative medicine that transplanted MSC do not necessarily engraft and differentiate at the site of injury [38]. Recent studies have shown that the key mechanism by which MSC contribute to tissue repair and regeneration is through their paracrine function [39, 40]. The present study found that the proliferation and type I collagen contents of fibroblasts from women with SUI increased after the fibroblasts were treated with adMSC-conditioned medium. Furthermore, we also found that under the influence of adMSC-conditioned medium, the expression levels of TIMP-1 and TIMP-3 were upregulated and the expression levels of MMP-1 and MMP-2 were downregulated. These results demonstrate that adMSC-conditioned medium regulated collagen metabolism in fibroblasts from women with SUI by increasing collagen synthesis and decreasing collagenolysis.

Recent studies have revealed that transplanted MSC may secrete abundant particles documented as exosomes, which are suggested to be central mediators of intercellular communication [41]. Since the first report of MSC-derived exosomes, a growing number of studies have explored their regenerative potential using different in vitro and in vivo models, and Hu et al. found that ASC-Exos could be taken up and internalized by fibroblasts to stimulate cell migration, proliferation and collagen synthesis [42]. In the present study, we successfully isolated and purified exosomes from human adMSC. In addition to demonstrating that Dil-labeled exosomes could be transferred to the cytoplasm of cultured fibroblasts from women with SUI, we further showed that the levels of type I collagen in cultured fibroblasts were significantly increased after the fibroblasts were treated with adMSC-Exos. We also found that adMSC-Exos upregulated the expression levels of TIMP-1 and TIMP-3 and downregulated the expression levels of MMP-1 and MMP-2. These results were consistent with those from adMSC-conditional medium, suggesting that adMSC-Exos play a crucial role in regulating collagen metabolism in fibroblasts from women with SUI.

## Conclusions

To our knowledge, this is the first study demonstrating the modulation of collagen metabolism in fibroblasts from women with SUI by adMSC-Exos. Because exosomes are easier to preserve and transfer and have low immunogenicity, they are safe for therapeutic administration. Therefore, the use of adMSC-Exos provides an efficient and safe alternative to mesenchymal stem cell-based therapies in the treatment of SUI in the future.

## Abbreviations

adMSC-CM: Adipose-derived mesenchymal stem cell-conditioned medium
adMSC-Exos: Exosomes secreted by adipose-derived mesenchymal stem cells
ALP: Alkaline phosphatase
MMP-1: Matrix metallopeptidase 1
MMP-2: Matrix metallopeptidase 2
MMP-9: Matrix metallopeptidase 9
MSC: Mesenchymal stem cells
SUI: Stress urinary incontinence
TIMP-1: TIMP metallopeptidase inhibitor 1
TIMP-2: TIMP metallopeptidase inhibitor 2
TIMP-3: TIMP metallopeptidase inhibitor 3
